# How Synchronization Protects from Noise

**DOI:** 10.1371/journal.pcbi.1000637

**Published:** 2010-01-15

**Authors:** Nicolas Tabareau, Jean-Jacques Slotine, Quang-Cuong Pham

**Affiliations:** 1 LPPA, Collège de France, Paris, France; 2 INRIA, École des mines de Nantes, France; 3 Nonlinear Systems Laboratory, Massachusetts Institute of Technology, Cambridge, Massachusetts, United States of America; The University of New South Wales, Australia

## Abstract

The functional role of synchronization has attracted much interest and debate: in particular, synchronization may allow distant sites in the brain to communicate and cooperate with each other, and therefore may play a role in temporal binding, in attention or in sensory-motor integration mechanisms. In this article, we study another role for synchronization: the so-called “collective enhancement of precision”. We argue, in a full nonlinear dynamical context, that synchronization may help protect interconnected neurons from the influence of random perturbations—intrinsic neuronal noise—which affect all neurons in the nervous system. More precisely, our main contribution is a mathematical proof that, under specific, quantified conditions, the impact of noise on individual interconnected systems and on their spatial mean can essentially be cancelled through synchronization. This property then allows reliable computations to be carried out even in the presence of significant noise (as experimentally found e.g., in retinal ganglion cells in primates). This in turn is key to obtaining meaningful downstream signals, whether in terms of precisely-timed interaction (temporal coding), population coding, or frequency coding. Similar concepts may be applicable to questions of noise and variability in systems biology.

## Author Summary

Synchronization phenomena are pervasive in biology, creating collective behavior out of local interactions between neurons, cells, or animals. On the other hand, many of these systems function in the presence of large amounts of noise or disturbances, making one wonder how meaningful behavior can arise in these highly perturbed conditions. In this paper we show mathematically, in a general context, that synchronization is actually a means to *protect* interconnected systems from effects of noise and disturbances. One possible mechanism for synchronization is that the systems jointly create and then share a common signal, such as a mean electrical field or a global chemical concentration, which in turn makes each system directly connected to all others. Conversely, extracting meaningful information from average measurements over populations of cells (as commonly used for instance in electro-encephalography, or more recently in brain-machine interfaces) may require the presence of synchronization mechanisms similar to those we describe.

## Introduction

Synchronization phenomena are pervasive in biology. In neuronal networks [1]–[3], a large number of studies have sought to unveil the mechanisms of synchronization, from both physiological [4],[5] and computational viewpoints (see for instance [6] and references therein). In addition, the *functional* role of synchronization has also attracted considerable interest and debates. In particular, synchronization may allow distant sites in the brain to communicate and cooperate with each other [7]–[9] and therefore may play a role in temporal binding [10],[11] and in attention and sensory-motor integration mechanisms [12]–[14].

In this article, we study another role for synchronization: the so-called *collective enhancement of precision* (see e.g. [15]–[17]), an intuitive and often quoted phenomenon with comparatively little formal analysis [18]. We explain mathematically why synchronization may help *protect* interconnected nonlinear dynamic systems from the influence of random perturbations. In the case of neurons, these perturbations would correspond to so-called “intrinsic neuronal noise” [19], which affect all of the neurons in the nervous system. In the presence of significant noise intensities (as experimentally found in e.g. retinal ganglion cells in primates [20]), this property would be required for meaningful and reliable computations to be carried out.

It should be noted that “protection of systems from noise” and “robustness of synchronization to noise” are two different concepts. The latter concept means that the synchronized systems remain so in presence of noise, whereas the former concept means that, thanks to synchronization, the behaviors of the coupled systems are close to the noise-free behaviors. This difference is further addressed in the Discussion.

**Figure 1:**
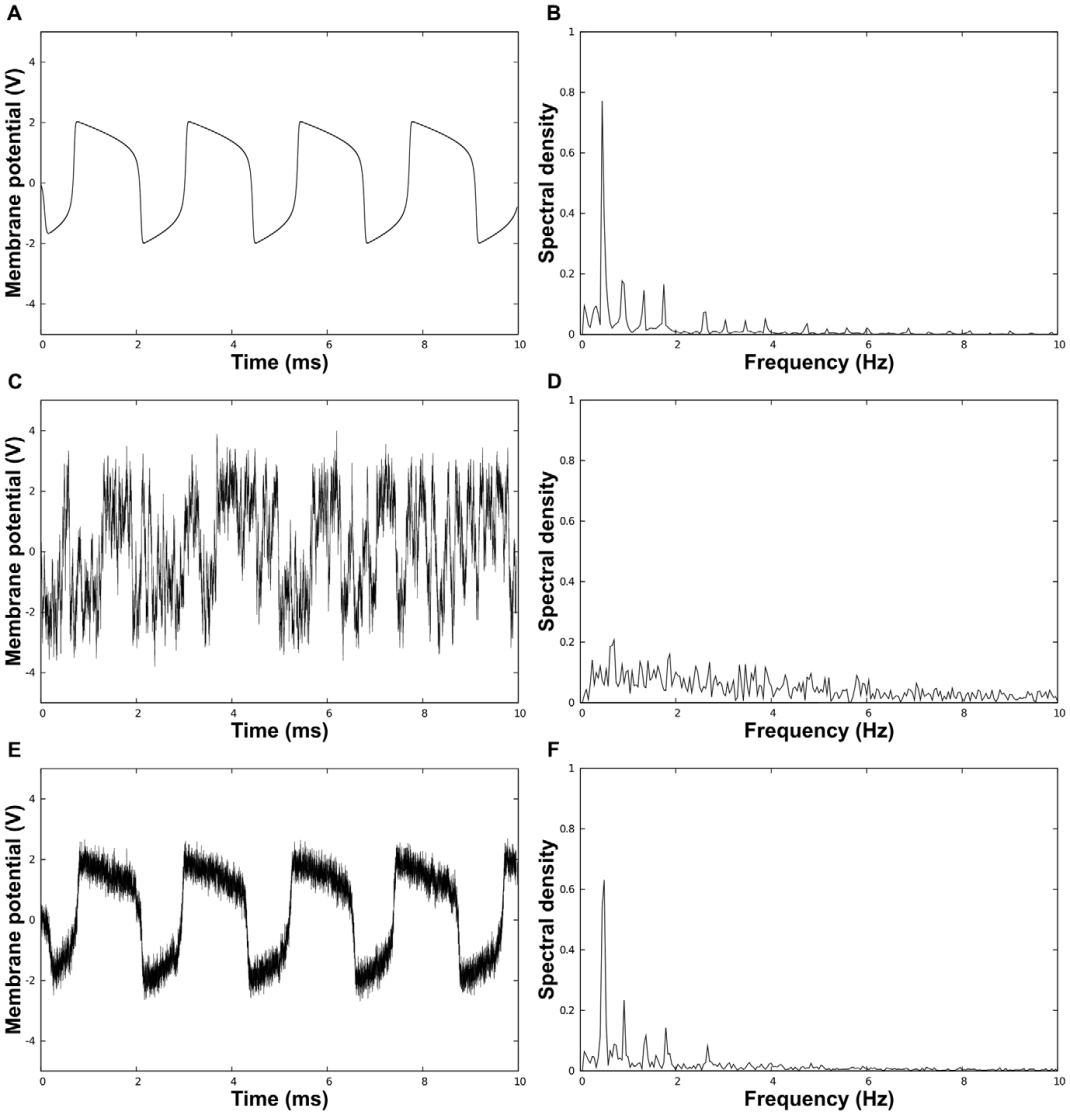
Simulations of a network of FN oscillators using the Euler-Maruyama algorithm [47]. The dynamics of coupled FN oscillators are given by equation (2). The parameters used in all simulations are 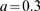, 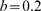, 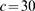. (A) shows the trajectory of the “membrane potential” of a noise-free oscillator and (B) depicts the frequency spectrum of this trajectory computed by Fast Fourier Transformation. (C) and (D) present the trajectory (respectively the frequency spectrum) of a *noisy uncoupled* oscillator (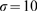). (E) and (F) show the trajectory (respectively the frequency spectrum) of a *noisy synchronized* oscillator within an all-to-all network (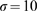, 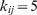, 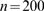). Note the temporal and frequential similarities between a noise-free oscillator and a noisy synchronized one. For instance, the main frequency and the first harmonics are very similar in the two frequency spectra. In contrast, the frequency spectrum of a noisy uncoupled oscillator shows no clear harmonics.

The influence of noise on the behaviors of nonlinear systems is very diverse. In chaotic systems, a small amount of noise can yield dramatic effects. At the other end of the spectrum, the effect of noise on nonlinear *contracting* systems is bounded by 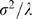 where 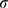 is the noise intensity – which can be arbitrarily large – and 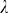 is the contraction rate of the system [21]. Between these two extremes, it has been shown analytically that some limit-cycle oscillators commonly used as simplified neuron models, such as FitzHugh-Nagumo (FN) oscillators, are basically unperturbed when they are subject to a small amount of white noise [22]. Yet, a larger amount of noise breaks this “resistance”, both in the state space and in the frequency space [Figures 1(A)–(D)]. This suggests that both temporal coding and frequency coding may be unusable in the context of large neuronal noise.

**Figure 2:**
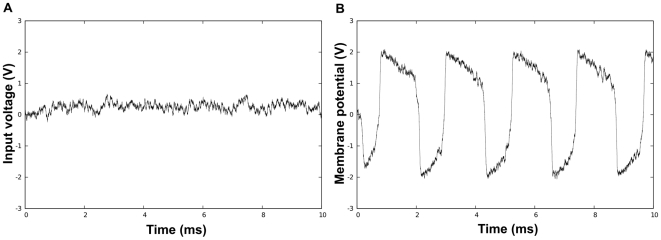
“Spatial mean” of FN oscillators. Note that the same set of random initial conditions was used in the two plots. (A) shows the average “membrane potential” computed over 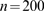 *noisy uncoupled* oscillators (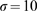). (B) shows the average “membrane potential” computed over 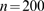 *noisy synchronized* oscillators within an all-to-all network (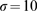, 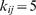). Observe that, in the first plot, the average trajectory of uncoupled oscillators carries essentially no information, while in the second plot, the average trajectory of synchronized oscillators is very similar to a noise-free one.

One might argue that it could be possible to recover some information from the noisy FN oscillators by considering the activities of a large number of oscillators *simultaneously* [19],[23]. Figure 2(A) shows that the spatial mean of the noisy oscillators still carries very little information when the noise intensities are large, making the population coding hypothesis also unlikely in this context. In other words, if the underlying dynamics are fundamentally *nonlinear*, as in the case of our FN oscillators, the spatial mean of the signals is “clean,” but contains very little information: the nonlinear nature of the systems dynamics prevents the familiar “averaging out” of noise through multiple measurements, and getting rid of the noise also gets rid of the signal.

By contrast, one can observe that when oscillators are *synchronized* through mutual couplings, then they become “protected” from noise, whether in temporal [Figure 1(E)], frequential [Figure 1(F)] or “populational” aspects [Figure 2(B)]. Thus, in some sense, the linear effect of averaging noise while preserving signal [24] can be achieved for these highly nonlinear dynamic components *through the process of synchronization*. Our aim in this article is to give mathematical elements of explanation for this phenomenon, in a full nonlinear setting. It is also to suggest elements of response to a more general question, namely: what is the precise *meaning* of ensemble measurements or population codes, and what information do they convey about the underlying dynamics and signals?

## Results

### General analytical result

Consider a diffusive network of 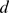-dimensional noisy non-linear dynamical systems
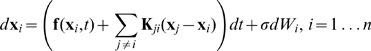
(1)where 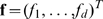 is a 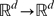 function. Note that the noise intensity 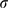 is intrinsic to the dynamical system (i.e. independent of the inputs), which is consistent with experimental findings [20]. For simplicity, we set 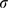 to be a constant in this article, although the case of time- and state-dependent noise intensities can be easily adapted from [21].

We consider four mathematical assumptions that will enable us to relate the trajectory of any noisy element of the network 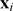 to the trajectory of the noise-free system 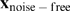 driven by equation
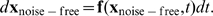
(A1) is an assumption on the form of the network. (A2) gives a bound on the nonlinearity of the dynamics 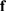. (A3) states that the system trajectories are resistant to small perturbations. Finally, (A4) requires that the dynamical systems in the network are synchronized.

#### (A1)

The network is balanced, that is, for any element of the network, the sum of the incoming connection weights equals the sum of the outgoing connection weights
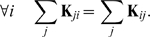
Remark that any symmetric network is balanced.

A particular kind of balanced network consists of an all-to-all network with identical couplings, i.e. 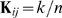 for all 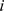 and 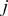. In general, assuming all-to-all coupling needs not be unduly restrictive, since such coupling can be implemented through mechanisms such as *quorum sensing* [25]–[27]. Indeed, assuming that the mean value of the 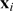
's can be provided by the environment as 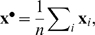 then the all-to-all network (1) can be written as a star network where damping is added locally and each cell 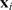 is only connected to the common signal
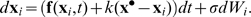
Quorum sensing, and more generally the measurement of a common mean signal, can thus be seen as a practical (and biologically plausible) way to implement all-to-all coupling with 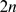 connections instead of 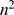.

#### (A2)

Let 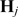 denote the Hessian matrix of the function 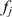 and let 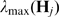 denote its largest eigenvalue. For all 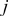, we assume that 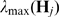 is uniformly upper-bounded by a constant 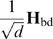. This implies in particular that
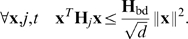
This assumption gives us a bound on the nonlinearity of 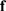, the extreme case being 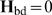 for a linear system.

#### (A3)

The dynamics 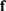 is resistant to small perturbations. More precisely, consider two systems starting from the same initial conditions but driven by slightly different dynamics
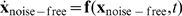
and
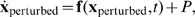
(where 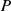 is a real time stochastic process) then 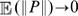 implies 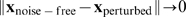.

In particular, such a property has been demonstrated in the case of FN oscillators, with 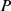 representing a white noise process [22].

#### (A4)

After exponential transients, the expected sum of the squared distances between the states of the elements of the network is bounded by a constant 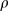


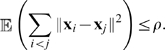
This is where synchronization will come into play, because synchronization is an effective way to reduce the bound 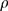. Some precise conditions for this will be given later.

We show in **Methods** that under these assumptions and when 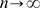 and 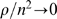, the distance between the trajectory of any noisy element 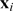 of the network and that of the noise-free system 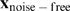 tends to zero, with the impact of noise on the mean trajectory evolving as
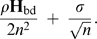
In particular, when 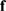 is a time-varying linear system of the form 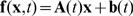, we recover the known result [28] that the impact of noise evolves as the inverse square root of 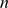. More generally, linear components of the system dynamics (including, in particular, the input signals) do not contribute to the first term of the above upper bound.

### Synchronization in networks of noisy FN oscillators

**Figure 3:**
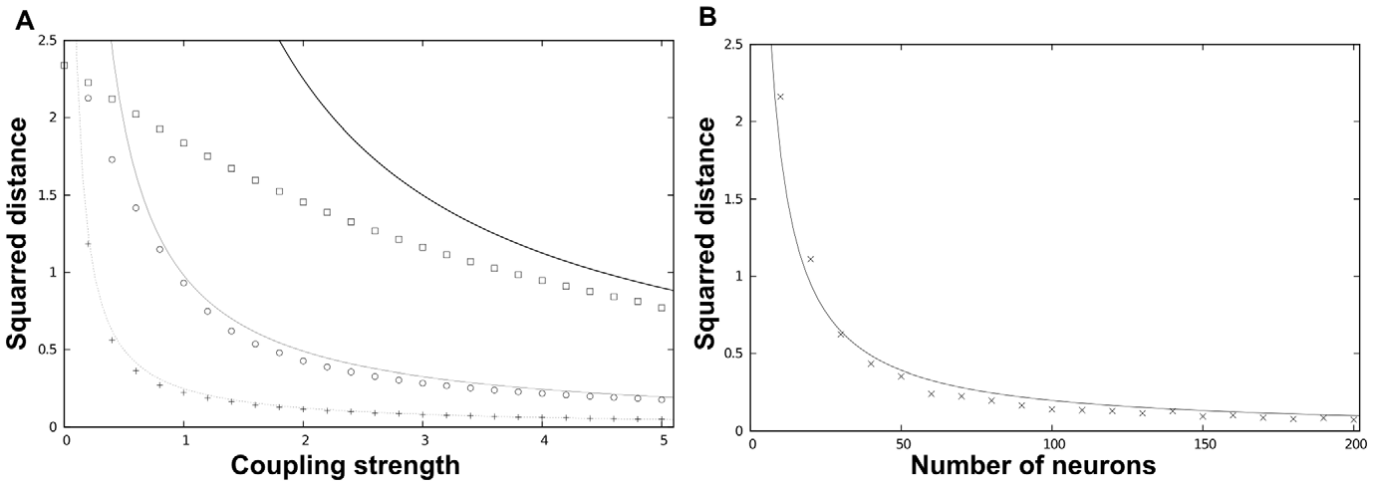
Asymptotic appraisal of the theoretical bounds. Note that the experimental expectations were computed assuming the ergodic hypothesis. (A) Expectation of the average squared distance between the 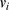
's and 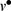 (given by 
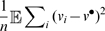
) as a function of the coupling strength 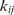 (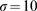). Theoretical bound 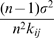 (cf equations (7) and (4)) for 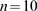 (bold line), for 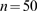 (plain line), for 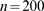 (dashed line); simulation results for 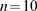 (squares), for 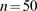 (triangles), for 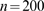 (crosses). (B) Expected squared distance between a noisy synchronized oscillator and its observer (given by 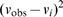) as a function of 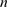 (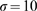, 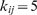). The bound 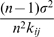 was plotted in plain line and the simulation results were represented by crosses.

We now give conditions to guarantee assumption (A4) for all-to-all networks of FN oscillators with identical couplings. The dynamics of 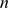 noisy FN oscillators coupled by (gap-junction-like) diffusive connections is given by
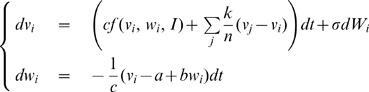
(2)where 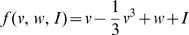. We show in **Methods** that, after exponential transients of rate 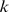,
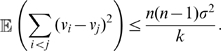
(3)Thus, (A4) is verified with
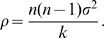
(4)For large 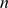, we have 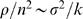, which converges to 0 when 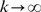. Figure 3(A) provides a comparison of this theoretical bound with simulations.

Assumption (A1) is also verified because an all-to-all network with identical couplings is symmetric, therefore balanced. Since the 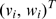 are oscillators with stable limit cycles, it can be shown that the trajectories of the 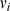 are bounded by a common constant 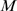. Thus (A2) is verified with 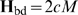. Finally, (A3) may be adapted from [22]. Indeed, we believe that the arguments of [22] can be extended to the case of non-white noise. Making this point precise is the subject of ongoing work.

Using now the “general analytical result”, we obtain that, given any (non necessarily small) noise intensity 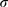, in the limits for 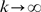 and 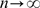 and after exponential transients, the behavior of any oscillator will be arbitrary close to that of a noise-free oscillator (Figure 1).

This statement can be further tested by constructing a model-based nonlinear state estimator (observer) [29]. Let 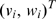 be a noisy synchronized oscillator and consider the observer
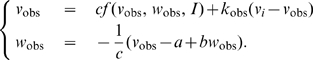
(5)


If 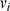 has the same trajectory as a noise-free FN oscillator, then it can be shown that 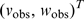 tends exponentially to 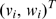, independently of the observer's initial conditions [29]. Thus the squared distance 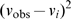 indicates how close 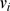 is from a noise-free oscillator [see Figure 3(B) for a comparison this theoretical result with simulations].

### Simulations of more generic networks

We provide in this section simulation results which show that similar observations can be made even for more general network classes that are not yet covered by the theory. We believe that this simulations show the genericity of the concepts presented above.

#### Probabilistic networks

**Figure 4:**
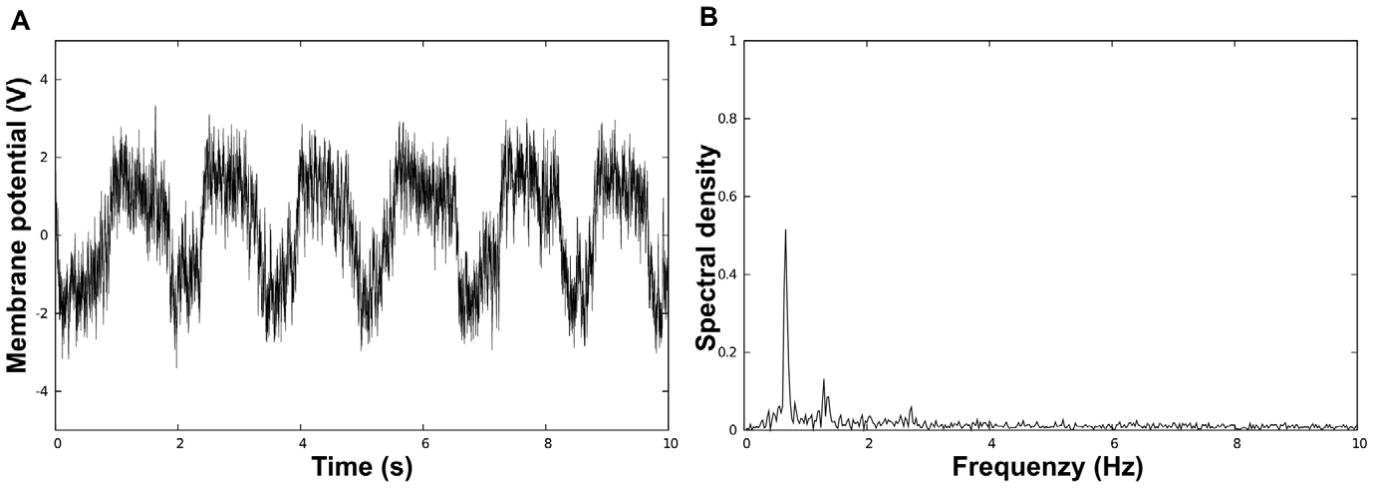
Simulation for a probabilistic symmetric network (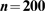, 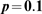, 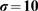, 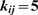). (A) shows the trajectory of the “membrane potential” of an oscillator in the network. (B) shows its frequency spectrum. Compare these two plots with those in Figure 1.

In practice, all-to-all neuronal networks of large size are rare. Rather, the mechanisms of neuronal connections in the brain are believed to be probabilisitic in nature (see [30] for a review). Here, we consider a probabilistic symmetric network of 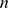 oscillators, where any pair of oscillators has probability 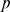 to be symmetrically connected and probability 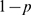 to be unconnected. Figure 4 shows simulation results for randomly chosen network with 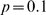. Concretely, we have built a network by randomly deciding for any pair of connections if the connection exists or not.

#### Hindmarsh-Rose oscillators

Hindmarsh-Rose oscillators are three-dimensional dynamical systems that are also often used as neuron models
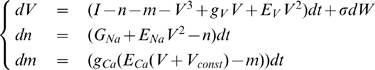
with 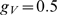; 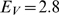; 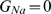; 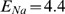; 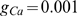; 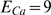; 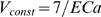. These oscillators can exhibit more complex behaviors (including spiking and bursting regimes [31]) than FitzHugh-Nagumo oscillators. The proofs of (A3) and (A4) for Hindmarsh-Rose oscillators are the object of ongoing research.

We made the inputs time-varying in this simulation. In fact, all the previous calculations can be straightforwardly extended to the case of time-varying inputs, as long as those inputs are the same for all the oscillators [6].

**Figure 5:**
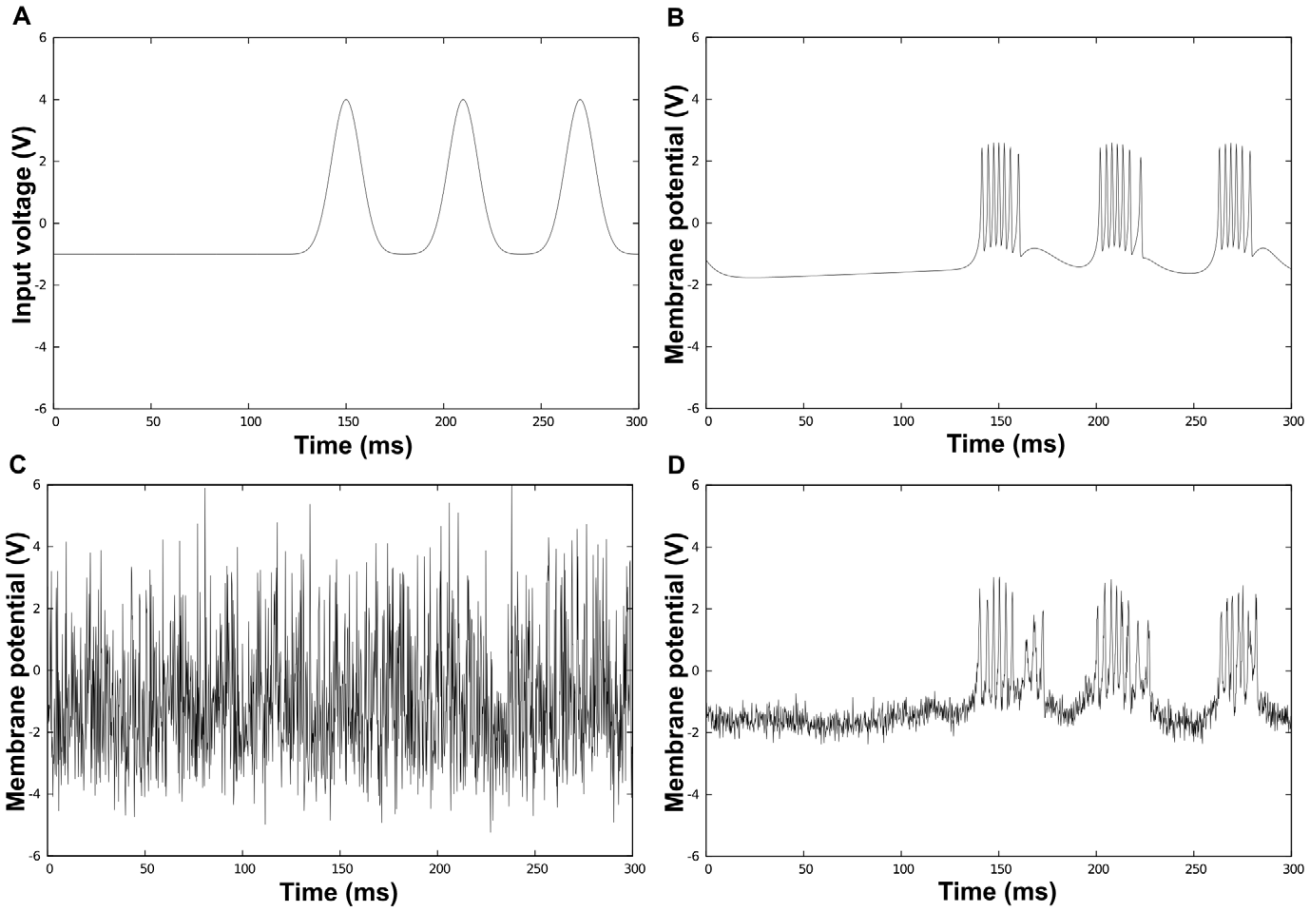
Simulation of Hindmarsh-Rose oscillators with time varying inputs. (A) The time-varying input voltage. (B) Trajectory of the “membrane potential” of a noise-free oscillator. (C) Trajectory of a *noisy uncoupled* oscillator. (D) Trajectory of a *noisy synchronized* oscillator (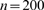, 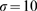, 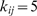).

One can observe from the simulations (see Figure 5) that the synchronized oscillators preserve the input signal, while the uncoupled oscillators completely blur it out.

## Discussion

We have argued that synchronization may represent a fundamental mechanism to protect neuronal assemblies from noise, and have quantified this hypothesis using a simple nonlinear neuron model. This may further strengthen our understanding of synchronization in the brain as playing a key functional role, rather than as being mostly an epiphenomenon.

It should be noted that the causal relationship studied here – effect of synchronization on noise – is converse to one usually investigated formally in the literature – effect of noise on synchronization: under certain conditions, adding noise can de-synchronize already synchronized oscillators (destructive effect) [32]; under other conditions, adding noise can, on the contrary, synchronize oscillators that were not synchronized (constructive effect) [33],[34]; for a review, see [35]. Also, previous papers have studied a similar phenomenon of improvement in precision by synchronization. Enright [28] shows 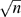 improvement in a model of coupled relaxation oscillators, all interacting through a common accumulator variable (possibly being the pineal gland). This 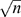 improvement has been experimentally shown in real heart cells [36]. More recently, [37] shows a way to get better than 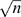 improvement. However, their studies primarily focused on the case of phase oscillators, which are linear dynamical systems. In contrast, we concentrate here on the more general case of nonlinear oscillators, and quantify in particular the effect of the oscillators' nonlinearities. The assumptions we consider are also different: while most existing approaches (including [37]) assume weak couplings and small noise intensities, we consider here strong couplings and arbitrary noise intensities.

The mechanisms highlighted in the paper may also underly other types of “redundant” calculations in the presence of noise and variability. In otoliths for instance, ten of thousands of hair cells jointly compute the three components of acceleration [38],[39]. In muscles, thousands of individual fibers participate in the control of one single degree of freedom. Similar questions may also arise in systems biology, e.g., in cell mechanisms of quorum sensing where individual cells measure global chemical concentrations in their environment in a fashion functionally similar to all-to-all coupling [25]–[27], in mechanical coupling of motor proteins [40], in the context of transcription-regulation networks [41],[42], and in differentiation dynamics [43].

Finally, the results point to the general question: what is the precise meaning of ensemble measurements or population codes, what information do they convey about the underlying signals, and is the presence of synchronization mechanisms (gap-junction mediated or other) implicit in this interpretation? As such, they may also shed light on a somewhat “dual” and highly controversial current issue. Ensemble measurements from the brain can correlate to behavior, and they have been suggested e.g. as inputs to brain-machine interfaces. Are these ensemble signals actually available to the brain [44], perhaps through some process akin to quorum sensing, and therefore functionally similar to (local) all-to-all coupling? Are local field potentials [45] plausible candidates for a role in this picture?

## Methods

### Proof of the general analytical result

In the noise-free case (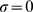), it can be shown that, for strong enough coupling strengths, the elements of the network synchronize completely, that is, after exponential transients, we have 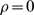 in (A4) [6]. Thus, all the 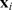 tend to a common trajectory, which is in fact a nominal trajectory of the noise-free system 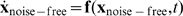, because all the couplings vanish on the synchronization subspace.

In the presence of noise, it is not clear how to relate the trajectory of each 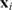 to a nominal trajectory of the noise-free system. Nevertheless, we still know that the 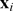 live “in a small neighborhood” of each other, as quantified by (A4). Thus, if the center of this small neighborhood follows a trajectory similar to a nominal trajectory of the noise-free system, then one may gain some information on the trajectories of the 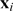.

To be more precise, let 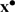 be the center of mass of the 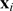, that is
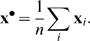
(6)Observe that, after expansion and rearrangement, the sum 
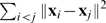
 can be rewritten in terms of the distances of the 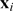 from 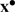


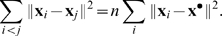
Using (A4) then leads to
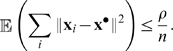
(7)Summing over 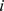 the equations followed by the 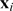 and using assumption (A1), we have
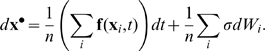
(8)We now make the dynamics explicit with respect to 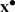 by letting
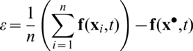
(9)so that Equation (8) can be rewritten as
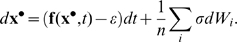
(10)Using the Taylor formula with integral remainder, we have
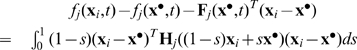
(11)where 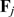 is the gradient of 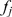 or, equivalently, the 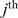 vector of the Jacobian matrix of 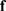. Summing Equation (11) over 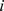 and using assumption (A2), we get
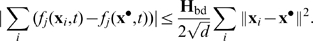
(12)Summing now inequality (12) over 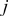 and using inequality (7), we get
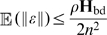
(13)which implies that 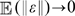 when 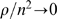.

Turning now to the noise term 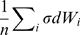 in Equation (10), we have
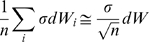
(14)since the intrinsic noises of the elements of the network are mutually independent.

Thus, for a given (even large) noise intensity 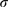, the difference between the *dynamics* followed by 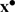 and the noise-free dynamics 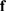 tends to zero when 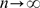 and 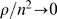. Assumption (A3) then implies that 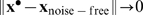. More precisely, the impact of noise on the mean trajectory (quantified by 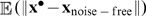) evolves as
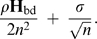
(15)Finally, Equation (7) and the triangle inequality
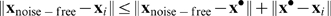
(16)imply that the *trajectory* of any synchronized element of the network 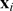 and that of the noise-free system 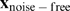 are also similar [compare Figure 1(A) and Figure 1(E)].

### FN oscillators in an all-to-all network

#### Two FN oscillators

Consider first the case of two coupled FN oscillators driven by Equation (2). Construct the following auxiliary system (or virtual system, in the sense of [46]), where 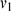 and 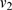 are considered as *external inputs*

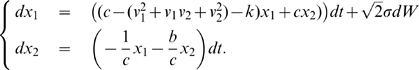
(17)Remark that 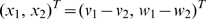 is a particular trajectory of this system.

Let 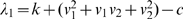 and 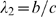. Assume that the coupling strength is significantly larger than the system's parameters, i.e. 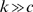, 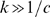 and 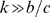. Since 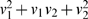 is nonnegative for any 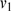 and 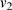, we have either 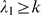 or 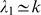, depending on the actual value of 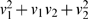. This implies in particular that 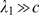, 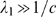 and 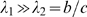.

Given these asymptotes, the evolution matrix of system (17) is diagonalizable with eigenvalues 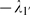 and 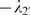 and eigenvectors respectively 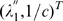 and 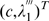. Furthermore, it is not difficult to see that all those 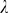
's are asymptotically close to each other, that is 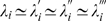 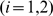. We now define
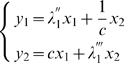
(18)leading to
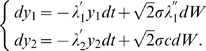
Since these equations are in fact uncoupled, they can be solved independently. Using the stochastic contraction results (corollary 1 of [21]) and the approximations 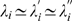, this yields
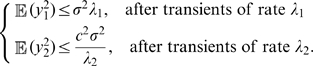
These bounds can be translated back in terms of the 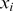 by inverting (18)
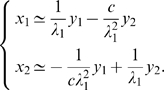
Thus, after transients of rate 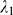,
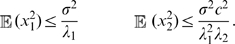
Since 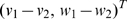 is a particular trajectory of system (17) as we remarked earlier, one finally obtains that, after transients of rate 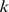,
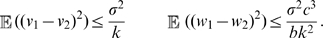
(19)


#### General case

Consider now an all-to-all network with identical couplings as in Equation (2). Construct as above the following 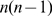 auxiliary systems indexed by 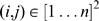, where the 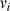 are considered as external inputs
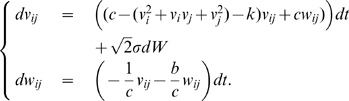
Remark that, similarly to the case of two oscillators, 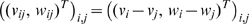 is a particular solution of these equations. Remark also that each pair 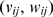 is in fact uncoupled with respect to other pairs. This allows us to use (19) to obtain that, after transients of rate 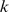,
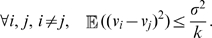
Summing over the 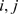 yields
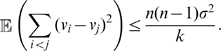
Thus, (A4) is verified with
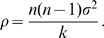
For large 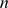, we have 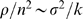, which converges to 0 when 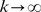 [see Figure 3(A)].

Assumption (A1) is also verified because an all-to-all network with identical couplings is symmetric, therefore balanced. As for (A2), observe that 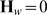 and
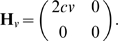
Since the 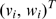 are oscillators with stable limit cycles, it can be shown that the trajectories of the 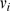 are bounded by a common constant 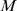. Thus (A2) is verified with 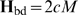.
